# Real‐Time Radiation Beam Monitoring by Flexible Perovskite Thin Film Arrays

**DOI:** 10.1002/advs.202401124

**Published:** 2024-08-28

**Authors:** Ilaria Fratelli, Laura Basiricò, Andrea Ciavatti, Lorenzo Margotti, Sara Cepić, Massimo Chiari, Beatrice Fraboni

**Affiliations:** ^1^ Department of Physics and Astronomy University of Bologna viale Berti Pichat 6/2 Bologna (BO) 40127 Italy; ^2^ National Institute for Nuclear Physics – Bologna viale Berti Pichat 6/2 Bologna (BO) 40127 Italy; ^3^ National Institute for Nuclear Physics – Florence Via G. Sansone 1 Sesto Fiorentino 50019 Florence Italy

**Keywords:** 2D hybrid perovskite, ionizing radiation direct detectors, large and flexible radiation detectors, proton detectors, transversal beam monitorin

## Abstract

Real‐time and in‐line transversal monitoring of ionizing radiation beams is a crucial task for several applications which span from medical treatments to particle accelerators in high energy physics. Here a flexible and large area device based on 2D hybrid perovskite thin films (phenylethylammonium lead bromide), fabricated onto a thin flexible polyimide substrate, able to map the transversal beam profile of high energy radiation beams is reported. The performance of this novel tool is here compared with the one offered by standard commercial large‐area technology, namely radiochromic sheets. The great potential of this class of devices is demonstrated by successfully mapping in real‐time a 5 MeV proton beam at fluxes between 10^8^ and 10^10^ H^+^ s^−1^ cm^−2^, confirming the capability to operate in a radiation‐harsh environment without output signal saturation issues. The versatility and scalability of here proposed detecting system are demonstrated by the development of a multipixel array able to map in real‐time a 40 kVp X‐ray beam spot (dose rate 8 mGy s^−1^). Perovskite thin film‐based detectors are thus assessed as a very promising class of thin, flexible devices for real‐time, in‐line, large‐area, conformable, reusable, transparent, and low‐cost transversal beam monitoring of different ionizing radiation.

## Graphical abstract

The transversal ionizing radiation beam monitoring by a 2D perovskite thin film‐based device is here reported for both 5 MeV protons and X‐rays. This novel class of detectors allows to develop a conformable, large area, low‐cost, low‐power supply, reusable, and transparent detecting system able to reconstruct the shape of different kinds of radiation beams.

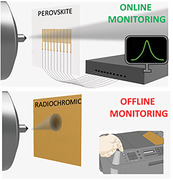

## 1 Introduction

Real‐time and in‐line monitoring of ionizing radiation beams play a crucial role in multiple application fields aiming to measure radiation field intensities, prevent eventual radiation damage issues, and avoid beam losses. Moreover, non‐destructive in‐line monitoring, i.e., interposing the detector between the radiation source and the final target without perturbing the radiation field, is a very desirable property in many circumstances. For instance, during radiation therapy treatments (e.g. radiotherapy or proton and hadron therapy) the precise delivery of the programmed dose to the patient and the online correction of misalignment issues are essential for optimal treatment effectiveness and for sparing the healthy tissues surrounding the tumor.^[^ ^1^ ^]^ The importance of real‐time monitoring has intensified with the emergence of cutting‐edge radiation therapy techniques, such as flash radiotherapy, where tens of Grays are delivered in few microseconds.^[^ ^2^, ^3^ ^]^

To meet the requirements for effective beam monitoring, several key factors must be addressed: i) high sensitivity is essential to detect low radiation doses, enabling precise measurements and dose calculations. The maximum uncertainty allowed in clinical operation is 2.5% on beam flux that can be guaranteed only if the detector provides high sensitivity,^[^ ^4^ ^]^; ii) a high signal‐to‐noise ratio (i.e., the dark current has to be lower than 1% of the signal current ^[^ ^5^ ^]^) is critical to distinguish the radiation signal from background noise and to ensure an accurate beam characterization, especially in the low doses range; iii) fast response signal (i.e., <1 ms) is necessary to capture dynamic changes in the radiation beam, providing real‐time monitoring capabilities. The readout frequency must exceed a few kHz to resolve the beam movements in the transverse plane that can be as fast as 100 mm ms^−1[^ ^4^ ^]^; iv) the detector should be transparent to the beam to avoid interference and to allow the accurate measurement of the beam profile even if it is interposed between the source and the final target; v) the detector must be tolerant to radiation to ensure long‐term stability and reliability (e.g. typically during cancer radiation treatments each dose fraction is ≈2 Gy); vi) a large area coverage (>10 × 10 cm^2^) is required for mapping the beam intensity onto large surfaces.

Nowadays available technologies, including diode arrays,^[^ ^6^, ^7^, ^8^, ^9^, ^10^, ^11^ ^]^ scintillating detectors,^[^ ^12^, ^13^ ^]^ ionization chambers (e.g. with either a single large electrode or electrodes segmented in strips or pixels or multi wires),^[^ ^14^, ^15^ ^]^ Faraday cups,^[^ ^16^ ^]^ microdiamonds,^[^ ^17^ ^]^ and radiochromic films,^[^ ^4^ ^]^ are able to only partially fulfill these requirements.

Among these technologies, radiochromic films are still considered a gold standard in beam monitoring tools, providing a high spatial resolution (0.1 – 0.2 mm) over large areas for different kinds of ionizing radiation beams.^[^ ^5^ ^]^ Tissue equivalence and ease‐of‐use are other appealing features of radiochromic films. However, the main limitation imposed by this technology, currently used in commercial densitometers and scanners, is the need of a post‐exposure processing to provide the received dose values, i.e., its inability to provide real‐time data. Only a few preliminary works recently proposed novel yet complex methods for their real‐time operation.^[^ ^18^ ^]^

Pixelated solid state detectors can combine the high spatial resolution offered by radiochromic films and the real‐time response offered by ionization chambers allowing immediate access to beam information without the need for time‐consuming and off‐line readout procedures. Yet, such devices are typically based on traditional Silicon/inorganic semiconductor technologies and thus absorb most of the impinging radiation, are rigid, and often require high power supply to operate. In the last decade, alternative material platforms such as organic semiconductors and lead‐halide perovskites emerged, demonstrating peculiar and very relevant properties. Several works reporting their good performances paved the path for the development of a novel class of thin and flexible detecting systems for different kinds of ionizing radiation (e.g., X‐rays,^[^ ^19^, ^20^, ^21^, ^22^, ^23^, ^24^, ^25^, ^26^, ^27^, ^28^ ^]^ protons,^[^ ^29^, ^30^, ^31^, ^32^ ^]^ neutrons,^[^ ^33^, ^34^, ^35^ ^]^). Organics and perovskites can be deposited from solution by means of low‐temperature and low‐cost fabrication processes. This property enables easy scalability to large and flexible substrates, making them adaptable to spatially map radiation beams onto curved surfaces (e.g., onto human bodies during a radiotherapy treatment, around circular pipelines of nuclear plants, or of nuclear accelerators) and for various beam monitoring setups. Furthermore, the patterning ability of these devices allows for envisaging pixelated beam monitors able to spatially map the radiation delivery.

Here, we report on how to take these devices a step further, investigating and discussing the employment of ionizing radiation dosimeters based on thin films of 2D layered hybrid perovskite phenylethylammonium lead bromide (PEA_2_PbBr_4_; PEA = C_6_H_5_C_2_H_4_NH_3_^+^) as much sought‐after real‐time and in‐line transversal beam monitoring devices. Thanks to the high sensitivities and good stability demonstrated by this semiconducting material,^[^ ^21^, ^36^ ^]^ the reduction of the pixel active volume (i.e., both pixel size and thickness) does not affect the reliability of the output signal. This allowed us to scale down the pixel dimensions, achieving a spatial resolution of 500 µm, and to employ thin active layers which assure an exceptional transparency to high energy radiation.

By exploiting the special features offered by this promising class of detectors, we were able to carry out accurate measurements of beam shape and spatial intensity distribution without significant beam attenuation and interference, successfully demonstrating the capability of real‐time and in‐line transversal mapping of both 5 MeV proton and 40 kVp X‐ray beams.

## 2 Results and Discussion

**Figure 1:**
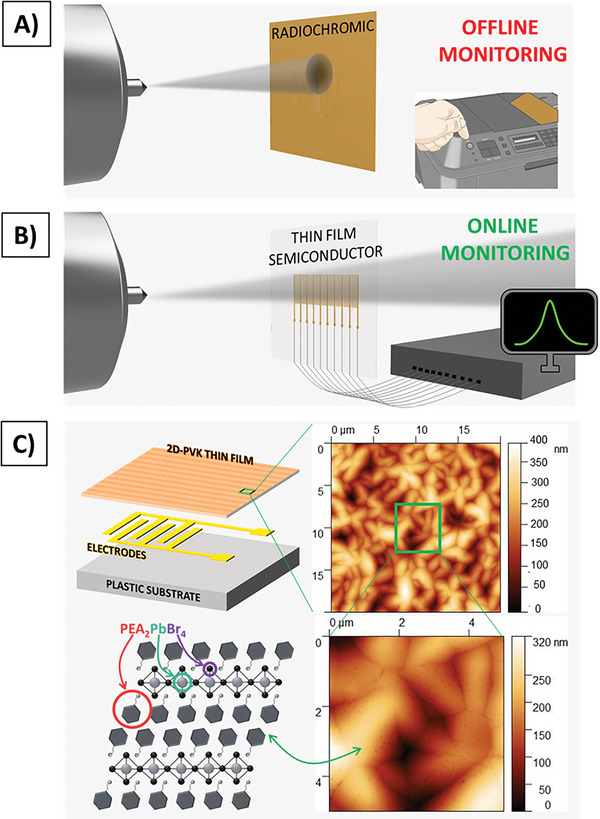
Sketch of radiation transversal beam monitoring acquired by A) commercial radiochromic film and B) semiconductor thin film‐based detector array. The first approach requires an off‐line analysis, while employing solid state detectors allows real‐time and online monitoring by the acquisition of the electrical signal instantaneously generated by the absorption of radiation. C) Schematic of perovskite thin film‐based detectors developed and investigated in this work and of the perovskite molecule employed as active layer. AFM images showing the morphology of the 2D perovskite thin film.

In this work, we developed and characterized solid‐state thin film‐based detectors for ionizing radiation beam monitoring based on hybrid perovskites and organic semiconductors. In **Figure** **1**a,b we illustrate the main advantages offered by these devices if compared with radiochromic foils. Radiochromic foils allow to determine the presence and intensity of ionizing radiation only via post‐irradiation analyses and cannot be re‐used. On the contrary, the here proposed solid‐state thin film detectors can monitor in real‐time the radiation delivered by the beam by simply and directly acquiring the output current signal and detecting tens of subsequent irradiation cycles (operating consecutively for over one hour), showing negligible degradation of the collected signal (see Figure S1, Supporting Information). Another characteristic offered by this class of detectors is related to their low thickness (in the 0.1 – 10 µm range) and low density (in the 1 – 2 g cm^−3^ range). Despite this aspect providing a lower extrinsic efficiency of the detector because of the lower attenuation factor, on the other hand, it offers a great advantage by means of a very low interference with the impinging radiation beam, a crucial requirement for an in‐line beam monitoring device. As will be shown in the following, these devices can be employed to detect different types of ionizing radiation (e.g., here we report X‐rays and protons), envisaging for a universal flexible and large area high energy radiation beam monitoring tool.

**Figure 2:**
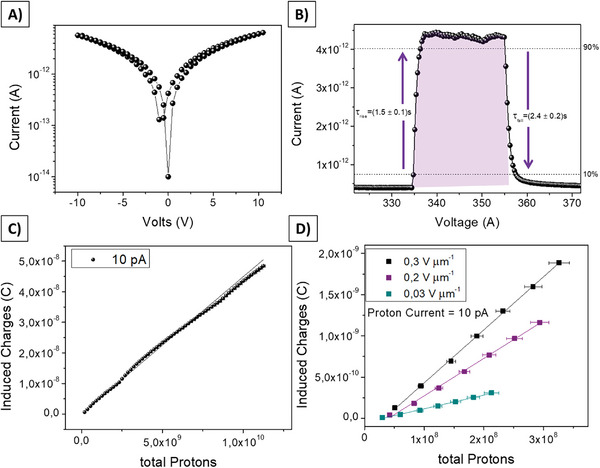
A) Current–Voltage curve of the 2D perovskite thin film‐based detector. B) Dynamic response of the detector polarized at 5 V (0.2 V µm^−1^) and irradiated by 5 MeV proton beams at (9.0 ± 0.2)·10^8^ H^+^ s^−1^ cm^−2^ (10 pA) of intensity. The absorption of energy from the proton beam provokes the increase of the current flowing in the device channel and the integral of the curve (pink shadow) indicates the proton‐induced charges collected at the electrodes. The two arrows indicate the rise time (τ_rise_ = (1.5 ± 0.1) s) and the falling time (τ_fall_ = (2.4 ± 0.2) s) calculated as the time for reaching the 90% of the signal starting from the 10% (i.e., the opposite for the falling time). C) Dose Linearity curve obtained by integrating at different instant times the peak reported in B). The plot confirms a very good linearity (R^2^ = 0.996) over more than two orders of magnitude of total proton number impinging onto the device. D) Dose linearity acquired polarizing the device with three different biases (voltages 1, 5, and 10V) which correspond to different electric fields 0.03, 0.2, and 0.3 V µm^−1^. The plot shows that the collected induced charges increase following the increase in the electric field (i.e., bias applied at the electrodes).

We employed a 2D hybrid perovskite thin film as the active semiconducting layer of the detector (i.e., PEA_2_PbBr_4_ (PEA = C_6_H_5_C_2_H_4_NH_3_^+^),^[^ ^21^ ^]^). The molecular structure of the perovskite is reported in Figure 1c. The film has been deposited from solution by spin coating on the top of two interdigitated gold electrodes obtaining a coplanar photoconductor architecture (see Figure 1c). The device has been realized onto a polymeric substrate (i.e., 500 µm thick polyimide) to guarantee the flexibility and the conformability of the detecting system. More details about the fabrication procedure are reported in the Experimental Section. The well‐packed thin film crystalizes as shown by the AFM images reported in Figure 1c. The film morphology is shown by the topographic and cross‐sectional SEM images reported in **Figure** **2** where the homogeneity of the thin film and the absence of pinholes are further demonstrated. The good crystallinity of the perovskite material is also demonstrated by the grazing‐incidence X‐ray diffraction (GI‐XRD) measurements, performed at the X‐ray Diffraction beamline 5.2 at the Synchrotron Radiation Facility Elettra in Trieste (Italy) (see Experimental Section for more details). By this experiment, reported in Figure S3 (Supporting Information), we assessed a good accordance between our pattern and the ones reported in literature.^[^ ^37^, ^38^ ^]^ We measured the average thickness of the film by AFM profile (see Supporting Information Figure S4, Supporting Information), which resulted 910 ± 50 nm.

In Figure 2 the electrical characterization under 5 MeV protons of a single pixel detector based on 2D perovskite is reported. The device presents the co‐planar architecture depicted in Figure 1c (channel length and width are 30 µm and 50 mm respectively, pixel area is 2 × 2 mm^2^). In Figure 2a the typical Current‐Voltage curve of the device is reported. The low hysteresis and electrical conductivity ((5.00 ± 0.01)·10^−3^ pS cm^−1^) are comparable with the values reported in literature,^[^ ^21^, ^36^ ^]^ confirming the high‐quality of the microcrystalline film. The device demonstrated a good environmental stability (see Figure S5, Supporting Information) showing no degradation after 4 months of storage in air, at room temperature, and in the dark. The high resistivity of the pure 2D perovskite active layer also lowers the dark current of about 2 orders of magnitude with respect to the 2D/3D mixed perovskite reported by our group previously,^[^ ^30^ ^]^ improving the Signal to Noise ratio of the detector (see Figure S6, Supporting Information).

The direct detector response under proton irradiation was tested using a 5 MeV beam extracted into ambient pressure, provided by the 3 MV Tandetron accelerator of the LABEC ion beam center (Laboratory of Nuclear Techniques for the Environment and Cultural Heritage, INFN Firenze, Italy).^[^ ^39^ ^]^ Proton beam currents used in this work are in the 1 – 100 pA range. The weak intensity of the beam is monitored and quantitatively measured using a rotating chopper,^[^ ^40^ ^]^ placed between the thin silicon nitride extraction window and the sample, that intercepts the beam. In Figure 2b an example of the current signal induced in the device due to the absorption of energy from a 5 MeV proton beam is reported. By Monte Carlo simulations (see Figure S7, Supporting Information) we calculated that each proton impinging onto the 2D perovskite layer passes through it and releases 12 keV µm^−1^ (i.e., 11 keV in the entire film). Here the device has been exposed to a 20 s irradiation cycle while it was kept at a bias voltage of 5 V (i.e., 0.2 V µm^−1^). The proton flux is (9.0 ± 0.2)·10^8^ H^+^ s^−1^ cm^−2^ and further details describing the irradiation protocols are reported in the Experimental Section. The absorption of energy from the protons provokes a steep increase in the current and the amount of charges induced by the radiation can be calculated as the integral of the curve (i.e., the pink shadow highlighted in Figure 2b). We calculated the rise and falling time as the time for reaching 90% of the signal starting from 10% (i.e., the opposite for the falling time). As it is shown in Figure 2b, the response times of the detector are τ_rise_ = (1.5 ± 0.1) s and τ_fall_ = (2.4 ± 0.2) s respectively. As shown in Figure 2c, the induced charges are proportional to the total number of protons impinging on the detector, showing a sensitivity of (4.25 ± 0.02)·10^−18^ C H^+ ‐1^ (i.e., the slope of the curve). Analogous plot achieved with a higher flux of protons, i.e., (1.2 ± 0.1)·10^10^ H^+^ s^−1^ cm^−2^, is reported in Figure S8 (Supporting Information). In this graph, each point represents the integral of the proton‐induced current at different times. In Figure 2d, the charges induced by protons and collected at the electrodes under three different polarization conditions (i.e., 0.03 V, 0.2, and 0.3 V µm^−1^) are reported as a function of the total number of impinging protons. The sensitivity value increases with the bias because of the improvement of collection efficiency with larger electric fields: (1.66 ± 0.05)·10^−18^, (4.25 ± 0.02)·10^−18^, and (6.43 ± 0.07)·10^−18^ C H^+ ‐1^ for 0.03, 0.2, and 0.3 V µm^−1^, respectively. As expected, the electrical noise increases at higher bias and consequently, the signal‐to‐noise ratio lowers for larger bias voltage conditions. By defining the Lowest Detectable Dose (LoD) of the detector as the intensity of radiation which induces a signal three times higher than the electrical noise,^[^ ^41^ ^]^ and under the assumption that noise is dominated by dark current shot noise and, the LoDs in the three bias conditions are (10.9 ± 0.4)·10^6^ H^+^, (6.1 ± 0.3)·10^6^ H^+^, and (2.3 ± 0.1)·10^6^ H^+^ for 0.3, 0.2, and 0.03 V µm^−1^ respectively. In order to keep a high sensitivity and a low LoD, all the measurements reported in this work have been carried out at 5 V bias voltage (i.e., 0.2 V µm^−1^).

The sensitivity value obtained in these polarization and irradiation conditions (up to S = (4.25 ± 0.02)·10^−18^ C H^+ ‐1^) is slightly higher than the performance we recently reported employing a 2D/3D mixed perovskite in the same device architecture (S = (1.12 ± 0.01)·10^−18^ C H^+ ‐1^),^[^ ^30^ ^]^ and two orders of magnitude higher than the results achieved by organic‐based planar photoconductors biased below 0.03 V µm^−1^ (S = (6.4 ± 0.2)·10^−20^ C H^+ ‐1^),^[^ ^29^ ^]^ (see Table S1 Supporting Information). The sensitivity here reported is comparable to the ones presented by a vertical photoconductor based on MAPbBr_3_ thick single crystal irradiated using 3 MeV protons and biased at 0.01 V µm^−1^ (S = (2.19 ± 0.03)·10^−18^ C H^+ ‐1^).^[^ ^42^ ^]^ Also, the sensitivity here achieved is higher than the performances provided by inorganic thermally evaporated CsPbCl_3_ thin film‐based devices irradiated with 100–228 MeV protons at higher fluxes (i.e., 1–10 nA) and biased at 2 V µm^−1^ (S = 4·10^−20^ C H^+ ‐1^),^[^ ^32^ ^]^ even taking into account the different energy release due to the different proton energy ranges (i.e., LET for 100–228 MeV protons in perovskite thin film is one order of magnitude lower than for 5 MeV protons).

**Figure 3:**
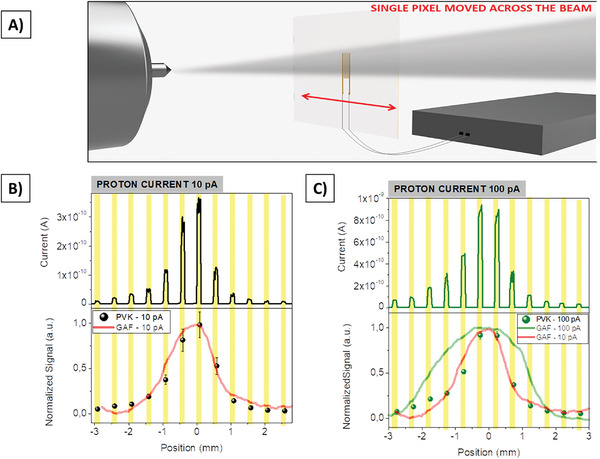
A) A perovskite (PVK) single pixel has been moved transversally in front of the extracted 5 MeV proton beam using a stepper motor (step size 0.5 mm). In each position, the response of the detector under two different proton currents has been acquired. Dynamic response (top) and normalized signal (bottom) recorded for the proton beam at 10 pA B) and 100 pA C). The symbols represent the proton‐induced charges recorded at each position by the perovskite based‐detector while the lines correspond to the signal recorded by a commercial radiochromic foil in the same irradiation conditions (GAFCHROMIC – HD V2).

**Figure** **3** reports the characterization of the 2D perovskite‐based detector as in‐line and real‐time 5 MeV proton beam monitoring tool. Here we tested a single pixel device (channel length L = 30 µm, width W = 30 mm and the entire pixel area is 0.5 × 5 mm^2^) by moving it across the beam spot from one side to the other using a step motor (step size = 0.5 mm) (Figure 3a). More details about the experimental setup are reported in the Experimental Section. In Figure 3b,c the beam profile extracted for two different proton fluxes are reported (i.e., (9.0 ± 0.2)·10^8^ H^+^ s^−1^ cm^−2^ and (1.2 ± 0.1)·10^10^ H^+^ s^−1^ cm^−2^). For each position we irradiated the samples for 10 s and we extracted the charges induced by protons following the procedure described above. The dynamic response of the 2D PVK‐based detector is reported in the top panels of Figure 3b,c. The signals have been acquired keeping the devices polarized at 5 V (0.2 V µm^−1^). In the bottom panels, the spheres represent the normalized signal induced at each position by the protons while the solid lines show the beam profile, extracted after off‐line reading, by a radiochromic sheet GAFCHROMIC – HD V2 exposed to the same beam conditions of the perovskite detectors (details are reported in the Experimental Section). At the lowest proton flux there is a good agreement between the 2D perovskite‐based detector and the radiochromic sheet indicating a successful reconstruction of the beam profile. At higher fluxes, the experimental points provided by the perovskite‐based detector still match the profile acquired by the radiochromic at lower fluxes, while the new profile extracted by the radiochromic foil is much broader. In Figure S9a,b (Supporting Information) the SRIM (Stopping and Range of Ions in Matter,^[^ ^43^ ^]^) simulation for the two fluxes in radiochromic foil is shown. By normalizing the transversal trace of the events for the different number of protons coming from the source, we obtain identical beam profiles, confirming that different proton fluxes do not affect the beam shape (Figure S9c, Supporting Information). From the profiles reported in Figure 3c we can conclude that, after 10 s of exposure at high proton flux, the radiochromic foil clearly shows a saturation effect leading to a distortion of the beam shape while the 2D perovskite based‐detector maintains its full detection properties and provides a reliable performance. This observation assesses the great potential of this new class of detectors in radiation harsh environments (e.g. radiotherapy treatments, nuclear physics accelerators, space missions), where the fluxes of charged particles are large and often cause saturation effects in solid‐state detectors and radiochromic foils.

**Figure 4:**
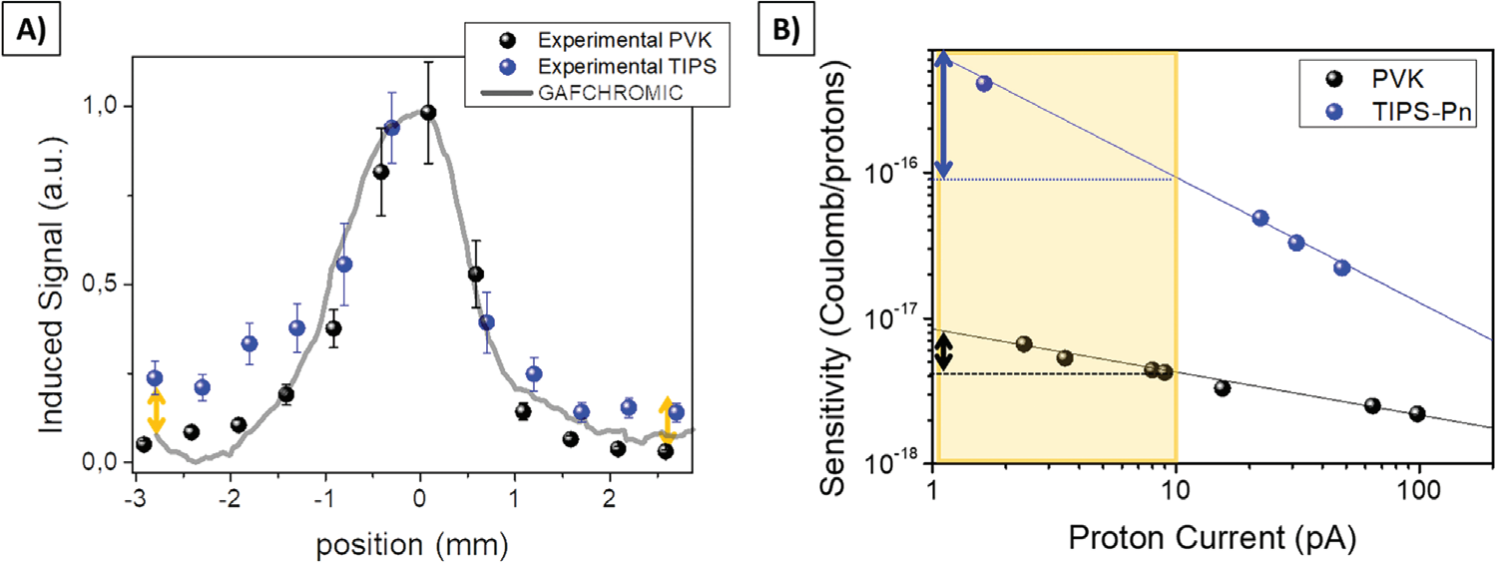
Comparison between the beam monitoring provided by the GAFCHROMIC – HD V2 (grey line), the 2D perovskite‐based detector (black symbols), and a TIPS‐pentacene based detector (blue symbols) sharing the same geometry and architecture. While the perovskite‐based detector allows to accurately monitor even the tails of the beam where the intensity of the radiation is lower, the organic based device does not reliably follow the shape of the beam in this region (yellow arrows are for eye guidance). B) Sensitivity values of the 2D perovskite‐based detector (black symbols) and a TIPS‐pentacene based detector (blue symbols) as a function of the proton current.

We also compared the performances of 2D perovskite‐based detectors with organic semiconductors‐based detectors, which represent an alternative promising class of low‐cost, large‐area, and flexible radiation detectors.^[^ ^19^, ^24^, ^28^, ^44^ ^]^ The organic semiconductor small molecule employed in this work is 6,13‐Bis(triisopropylsilylethynyl)pentacene (TIPS‐pentacene) deposited from solution by drop casting on the top of two interdigitated gold electrodes and fabricated onto 500 µm polyimide substrate. In **Figure** **4** we report the comparison of a 2D‐perovskite‐based detector and an organic semiconductor‐based one, sharing the same device architecture, for transversal proton beam monitoring. Figure 4a reports the two linescan of the proton beam spatial profile (proton current = 10 pA) achieved using the organic and 2D perovskite‐based detectors and compared with the nominal profile acquired by radiochromic film. The proton‐induced signal generated by the organic device under 5‐MeV protons at a flux of 10 pA is reported in Figure S10 (Supporting Information) and it is comparable to what has been reported previously by our group using a similar organic molecule and a different polymeric substrate.^[^ ^29^ ^]^ In general, low dimensional photoconductors where the detecting process is strongly ruled by a trap‐mediated inner mechanism of amplification called photoconductive gain (PG), often show a sublinear trend of the sensitivity as a function of the flux of the radiation. For devices based on such materials the gain factor G typically decreases with increasing radiation flux (i.e., for ionizing radiation dose rate, for visible photons light power), partly due to the gradually filled trap states which control the activation of PG effect. Once all the deep trap states are completely filled at a certain flux, a more intense radiation would excite more free carriers that cannot be trapped or can be trapped only by shallower defects. This results in a decrease of the average carrier lifetime and a consequent lowering of the PG activation effectiveness. For visible light, this effect is also known as dynamic‐range enhancing gain compression under increased illumination.^[^ ^45^ ^]^ Therefore, as it is reported in literature, typically at high fluxes of radiation, the gain is reduced as well as the sensitivity of the detector (i.e., responsivity in photodetector) as a consequence.^[^ ^45^, ^46^, ^47^ ^]^ This effect represents one of the main issues for dosimeters and radiation beam monitoring systems because it can lead to a severe distortion of the beam profile both in transversal and longitudinal configurations. It is well known that organic semiconducting thin films are defective materials,^[^ ^48^ ^]^ and the high density of electrical active traps makes them photoconductive gain‐driven detectors when they are implemented in a photoconductor architecture.^[^ ^49^ ^]^ On the contrary, lead‐halide perovskites are less defective materials. The limited number of trap states leads to a lower PG activation,^[^ ^50^ ^]^ and consequently to a lower dependency of the detecting response to the radiation flux, as shown in Figure 4b. This difference is clearly evidenced in Figure 4a where it is shows how the perovskite‐based detector can follow the shape of the proton beam also in the tails (i.e., where the flux of radiation is lower) while the organic‐based device over‐estimates the response leading to a distorted reconstruction of the beam shape.

**Figure 5:**
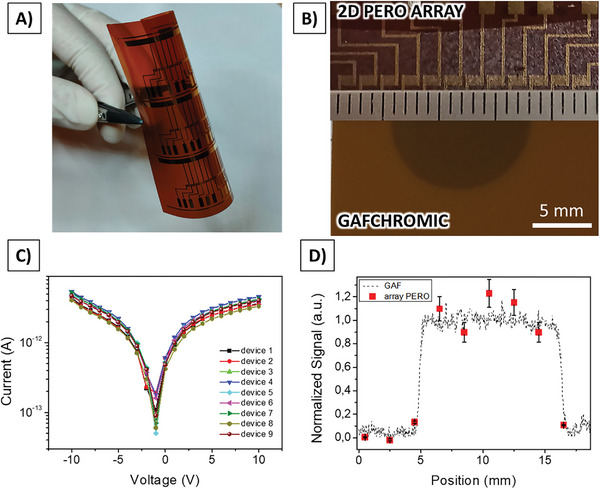
A) Scalability of 2D perovskites‐based detectors as pixelated and flexible beam monitoring devices. The planar perovskite photoconductor has been deposited as active layer for a 12‐pixel linear array fabricated onto a polyimide substrate. Here, a picture of three arrays fabricated in one single run is reported to demonstrate the scalability of the process. B) Linear 2D perovskite‐based array (top) and GAFCHROMIC sheet (XR‐QA2) (bottom) reporting a circular spot produced by an X‐ray beam (40 kVp, 500 µA, W‐target tube, 8 mGy s^−1^). C) IV curves of the pixels contained in the array necessary to map the beam spot. D) Normalized signal induced by the X‐rays provided by GAFCHROMIC (XR‐QA2) (line) and 2D perovskite array (symbol) show a good overlapping.

Finally, **Figure** **5** reports the proof of principle of the scalability of flexible 2D perovskite‐based detectors as large‐area beam monitoring tools. We fabricated multi‐pixels arrays to be employed for the in‐line and real‐time ionizing radiation beam monitoring. We tested these devices under X‐rays produced by a W‐target X‐ray tube (40 kVp, 500 µA, 8 mGy s^−1^). The full‐characterization of a single pixel device under X‐rays produced by the same experimental setup is reported in,^[^ ^21^ ^]^ and in Figure S11 (Supporting Information). As mentioned earlier, the hybrid 2D perovskite's low density and chemical composition contribute to minimal interference with the primary beam. However, this also results in a low attenuation fraction of high‐energy ionizing radiation, reflected in the measured X‐ray sensitivity value of S_A_ = 123 ± 2 nC Gy^−1^ cm^−2^. Besides, to demonstrate the mechanical flexibility of the here presented detecting system, we performed different tests under X‐rays while the 2D perovskite‐based detector was kept bent at different curvature radii down to R_C_ = 2 mm (see Figure S12, Supporting Information). These measurements demonstrate that the detecting response of the device decreased at 90% of the initial value acquired in the flat conditions but the original photocurrent is recovered once the device is placed back in the flat condition, indicating a reversable and not‐permanent effect induced by the mechanical stress.

Figure 5a shows three 12‐pixel arrays printed in one single run on a flexible polyimide substrate. Each pixel presents a co‐planar configuration similar to the one reported above, but with different dimensions resolution (L = 30 µm, W = 11 mm, pixel area is 1 × 1 mm^2^, pitch = 2 mm). Multiple arrays can be printed in one single run maintaining a very good yield (i.e., ≥90%) and demonstrating the easy scalability of the process even in a lab‐scale facility. In Figure 5b the picture of one single array is reported on top. On the bottom of the figure a radiochromic sheet (GAFCHROMIC XR‐QA2) simultaneously exposed to the same X‐ray beam spot is shown. Figure 5c reports the dark Current‐Voltage curves for 9 pixels from which we extracted the mean value of the electrical conductivity 6.3 ± 0.8 pS cm^−1^. In (Figure S13, Supporting Information) we also report the photocurrent peaks provided by the array exposed to X‐rays (40 kVp, 500 µA, W‐target tube, 8 mGy s^−1^). The variation of the pixel response is within 10%, demonstrating the excellent uniformity of the detecting performances achieved by the devices. Figure 5d, reports the actual experimental mapping of the X‐ray beam spot. For this test, both the 2D‐perovskite array and the radiochromic foil have been placed in front of the X‐ray tube aperture. The 2D perovskite‐based array has been connected to a custom multiplexing system for the readout. The red symbols represent the experimental points obtained by extracting and normalizing the photocurrent ΔI induced in 9 pixels of the array in different positions (ΔI = I_X‐Ray_ – I_dark_). The dashed line represents the profile of the spot acquired by the radiochromic sheet. It is noteworthy that we intentionally chose an X‐ray beam flux that would not induce saturation effects in the radiochromic foil, that we use here as a gold standard to validate the performance of the 2D‐perovskite array. The two curves are in excellent agreement, confirming a successful and reliable real‐time and in‐line transversal mapping of the X‐ray beam.

## 3 Conclusion

In this work, we report the performance of a 2D hybrid perovskite thin film‐based detector as a real‐time and in‐line transversal beam monitor for different types of ionizing radiation.

Three major results have been achieved: i) First, 2D perovskite‐based single pixel devices have been fully characterized as 5 MeV proton direct detectors and by moving them across radiation beam diameter we successfully reconstructed the proton beam profile under different flux irradiation conditions. We compared the response with radiochromic foils that are presently considered the gold standard for ionizing radiation beam monitoring. Notably, unlike radiochromic foils that exhibit saturation at higher proton fluxes, the 2D perovskite‐based device showed no saturation effects and it was able to accurately and reliably provide a transversal map of the beam shape and intensity distribution; ii) we compared the performance of 2D perovskite thin film detectors with organic semiconducting thin film‐based ones, and our findings indicate that the lower defect concentration and electrical activity of 2D perovskites with respect to organic thin films enable a more accurate reconstruction of the beam shape, even in regions with lower‐intensity radiation beams (i.e., tails of the beam); iii) we demonstrate the scalability of a flexible and large area beam monitoring system based on the here presented 2D perovskite‐based thin film devices as we implemented a multipixel arrays (12 pixels each) that we used to successfully and reliably monitor a 40kVp X‐ray beam shape.

In conclusion, our work demonstrates how to exploit the excellent capabilities recently reported for 2D perovskite thin film‐based detectors. Such flexible, large‐area, low‐cost, low‐power consumption, reusable, and transparent devices satisfy all the requirements for the implementation of a much sought‐after tool: a real‐time and in‐line ionizing radiation beam monitoring system.

## 4 Experimental Section

### Device Fabrication

The metal electrodes were fabricated on 500 µm thick polyimide substrate by lithographic techniques. Before electrode’ deposition, the substrates were cleaned by subsequent ultrasonic baths in H_2_O and soap, deionized H_2_O, and isopropyl alcohol. A positive photoresist (S1818) was then spin‐coated on the substrate at 4000 rpm for 60 s. The layout had been projected exposing the resist through an optical Microwriter (ML3Durham Magneto Optic). The resist had been developed by MICROPOSIT MF‐139 developer and then rinsed with deionized water. Electrodes were formed by 5 nm of Chromium and 45 nm of Gold thus deposited through thermal vacuum evaporation and patterned by dipping the whole structure into acetone bath for 4 h.

For the perovskite preparation, C_6_H_5_C_2_H_4_NH_3_Br (PEABr, Sigma‐Aldrich >98%) and PbBr_2_ (Sigma‐Aldrich >98%) were mixed inside a nitrogen filled glove box in N,N‐dimethylformamide (DMF, Sigma‐Aldrich 99.8% anhydrous) to prepare a 1 m solution with 2:1 molar ratio. The solution was mixed thoroughly for 5 h until complete dissolution of the precursors.

For the TIPS‐pentacene preparation, a 0.5%wt solution in toluene has been prepared.

The perovskite has been deposited by spin coating (800 rpm 10s + 2000 rpm 50s and 10 min of annealing at 60 °C), while the TIPS‐pentacene has been deposited by drop casting followed by a 1‐h thermal annealing at 90 °C.

### X‐Ray Irradiation

Characterization under X‐rays was performed using the X‐ray broad spectrum provided by a tungsten tube with an accelerating voltage of 40 kVp and tube current in the 10–500 µA range. These corresponded to dose rates in the 0.1‐8 mGy s^−1^ range. The multi‐pixel arrays had been placed orthogonal to the beam and connected to a custom multiplexing readout system.

### Proton Irradiation

The detectors were irradiated using a 5 MeV proton beam provided by the 3 MV Tandetron accelerator of the LABEC ion beam center (INFN Firenze, Italy). The beam was extracted into ambient pressure through a 200 nm thick Si_3_N_4_ membrane; the sample was typically mounted at a distance of 8 mm from the extraction window. Proton beam currents used in this work were typically in the 1 – 100 pA range. The weak intensity of the extracted beam was monitored and quantitatively measured using a rotating chopper, placed between the silicon nitride window and the sample, that intercepts the beam; the chopper was a graphite vane covered with a thin nickel evaporation, and the Ni X‐ray yield was used as an indirect measurement of the beam current.^[^ ^40^ ^]^

To determine the actual energy of the protons impinging onto the semiconducting layer, the energy lost by the protons passing through the several layers interposed between the beam and the sensor, namely, 200 nm of Si_3_N_4_ for the beam extraction window, 8 mm of mixed air‐He (50%–50%) atmosphere in the gap between the extraction window and the metal box, 13 µm of Al for the entrance window of the box, where the sensor was enclosed, and 36 mm of air inside the box, has to be calculated. After passing through these layers, protons lose ≈ 575 keV, as calculated with the SRIM Monte Carlo code.^[^ ^43^ ^]^

### GI‐XRD Measurements

GIXRD measurements were performed at the X‐ray Diffraction beamline 5.2 at the Synchrotron Radiation Facility Elettra in Trieste (Italy). For more detailed technical information see the work previously published by Basiricò et al.^[^ ^30^ ^]^

### SEM Images

The images were acquired by using a Cambridge Stereoscan 360 SEM, operating at 6 kV. The cross‐section samples were obtained by cutting the Kapton substrate at the edge and pulling apart two pieces by hand.

## Conflict of Interest

The authors declare no conflict of interest.

## Supporting information

Supporting Information

## Data Availability

The data that support the findings of this study are available from the corresponding author upon reasonable request.
