# Supplementary material for: Real‐Time Radiation Beam Monitoring by Flexible Perovskite Thin Film Arrays
Source: Adv Sci (Weinh). 2024 Aug 28;11(40):2401124. doi: 10.1002/advs.202401124 (PMC11516118; doi:10.1002/advs.202401124)
Supplement: Supplementary file 1 — Supporting Information [file ADVS-11-2401124-s001.pdf]

ADVANCED SCIENCE
Open Access

Supporting Information

for *Adv. Sci.*, DOI 10.1002/advs.202401124

Real-Time Radiation Beam Monitoring by Flexible Perovskite Thin Film Arrays

Ilaria Fratelli, Laura Basiricò, Andrea Ciavatti, Lorenzo Margotti, Sara Cepić, Massimo Chiari and Beatrice Fraboni*

Supporting Information

Real-time radiation beam monitoring by flexible perovskite thin film arrays

Ilaria Fratelli, Laura Basiricò*, Andrea Ciavatti, Lorenzo Margotti, Sara Cepić, Massimo Chiari, and Beatrice Fraboni

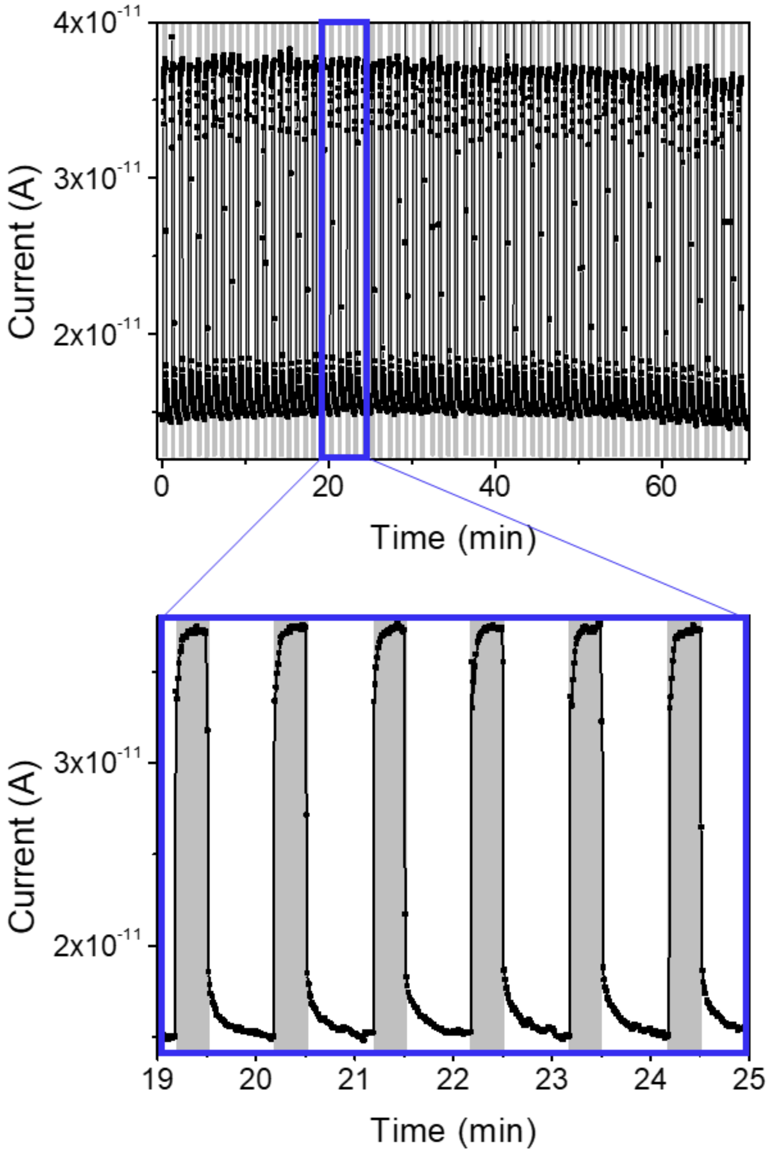

Figure S1. X-ray detection response under 70 subsequent irradiation cycles (20 s X-ray ON, 40 s X-ray OFF). The grey shadow indicated the time in which the X-rays are shining the sample. The X-rays are produced by a W-target tube kept at 40 kVp anode voltage and the dose rate is 8 mGy s^{-1}. The sample is polarized at 5 V (0.2 V μm^{-1}). The detecting signal is the difference between the current flowing in the channel when the radiation is ON and the dark current present before the turning ON of the X-rays. The photocurrent degradation after 1 hour of measurement (70 cycles) is 4%.

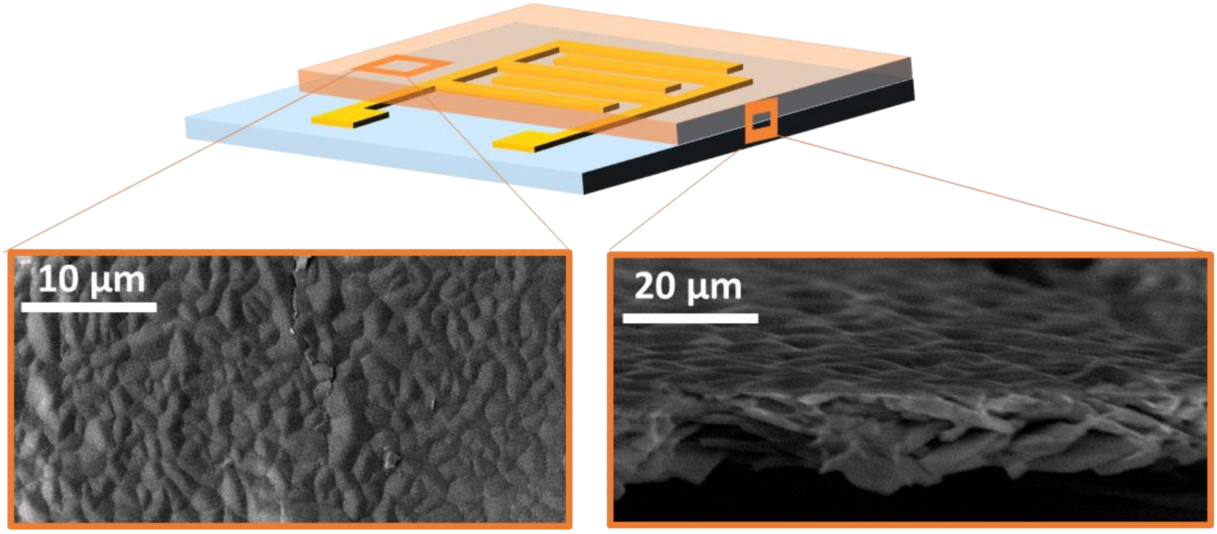

Figure S2. Topographic (left) and cross sectional (right) SEM images of the $(PEA)_2PbBr_4$ thin film.

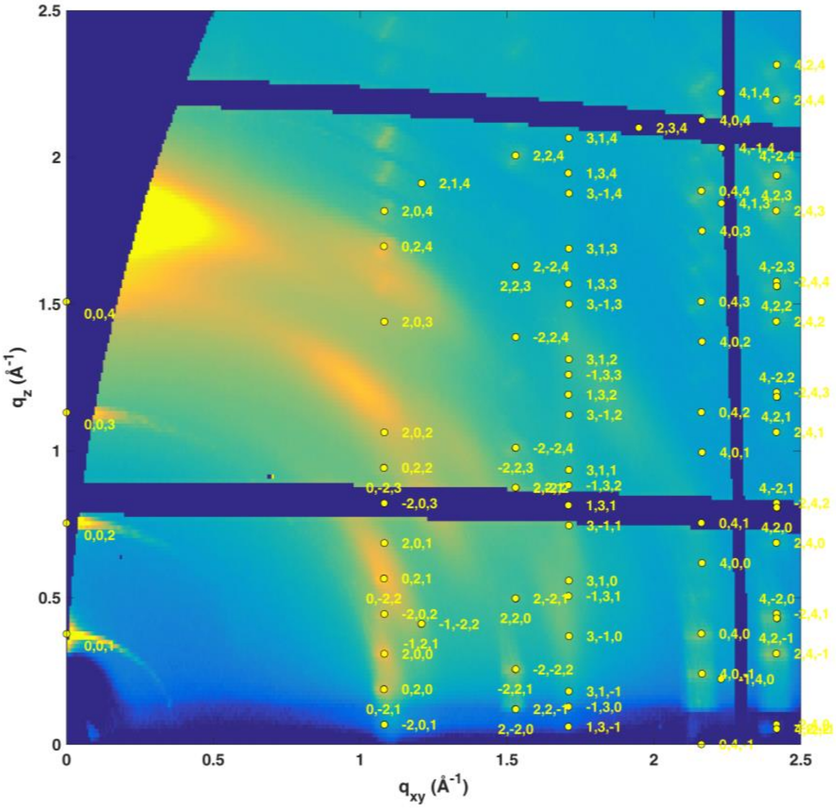

Figure S3 X-ray diffraction pattern in grazing incidence geometry of $(PEA)_2PbBr_4$ perovskite film, representing Log of diffracted intensity as a function of reciprocal lattice vector components q_{xy} and q_z. In the image the expected diffraction spots positions are reported together with indexes from the three components of the sample, oriented with c axis perpendicular to the sample surface.

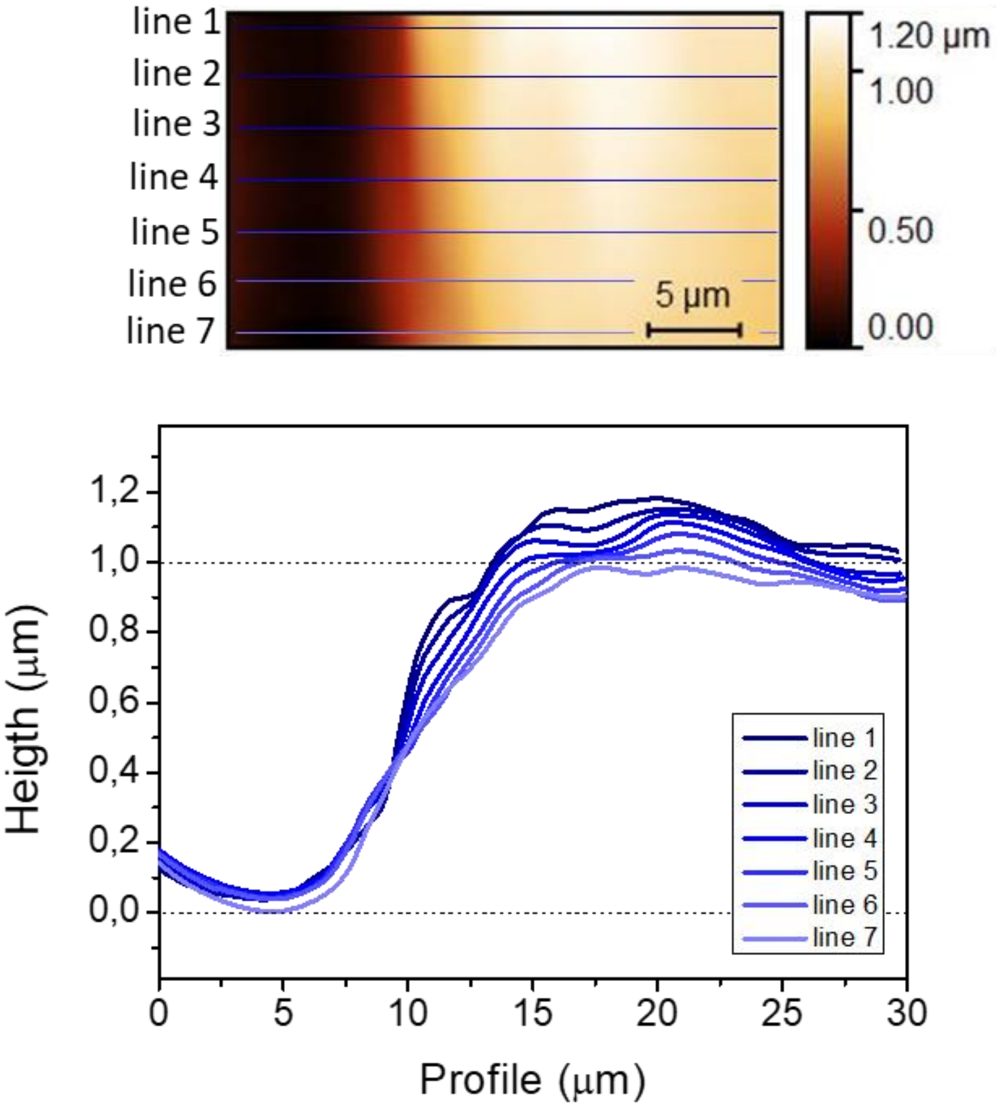

Figure S4. Measurement of the 2D perovskite film thickness. The thickness of the 2D perovskite active layer was assessed by making a scratch by means of a scalpel along the material film and acquiring an image of the area involved (3 µm x 30 µm) (up). The final thickness is calculated as the average value of seven different profiles extracted along the entire length of the groove (bottom).

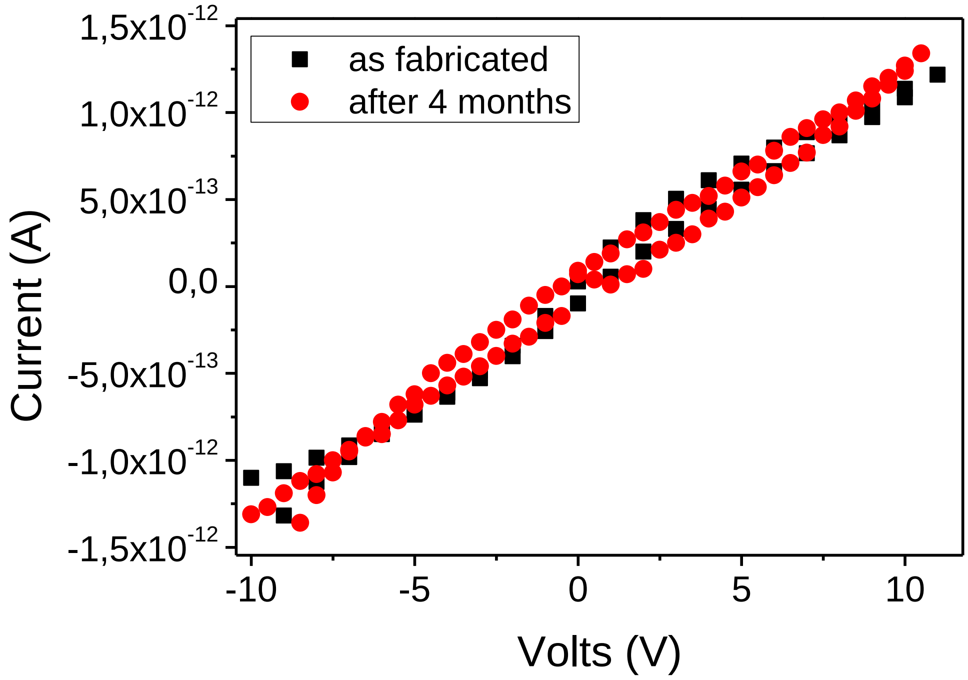

Figure S5. Current-Voltage curve of a 2D perovskite-based device acquired after the fabrication (black solid squares) and after four months of storage in air, at room temperature and in dark (red solid circles).

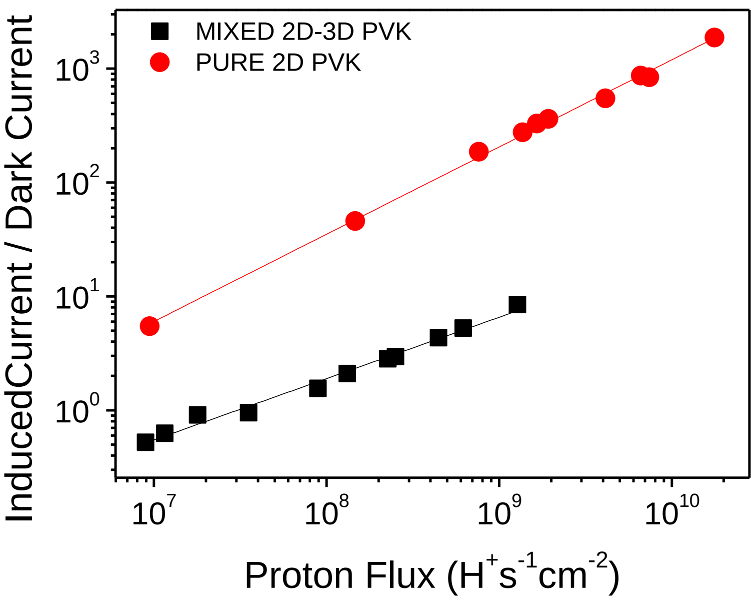

Figure S6. Induced Current by different proton fluxes normalized for the dark current flowing in the device without radiation. The dark current value for the 2D perovskite-based device is in the order of magnitude of 10^{-13} A while for the mixed is 10^{-11} A.

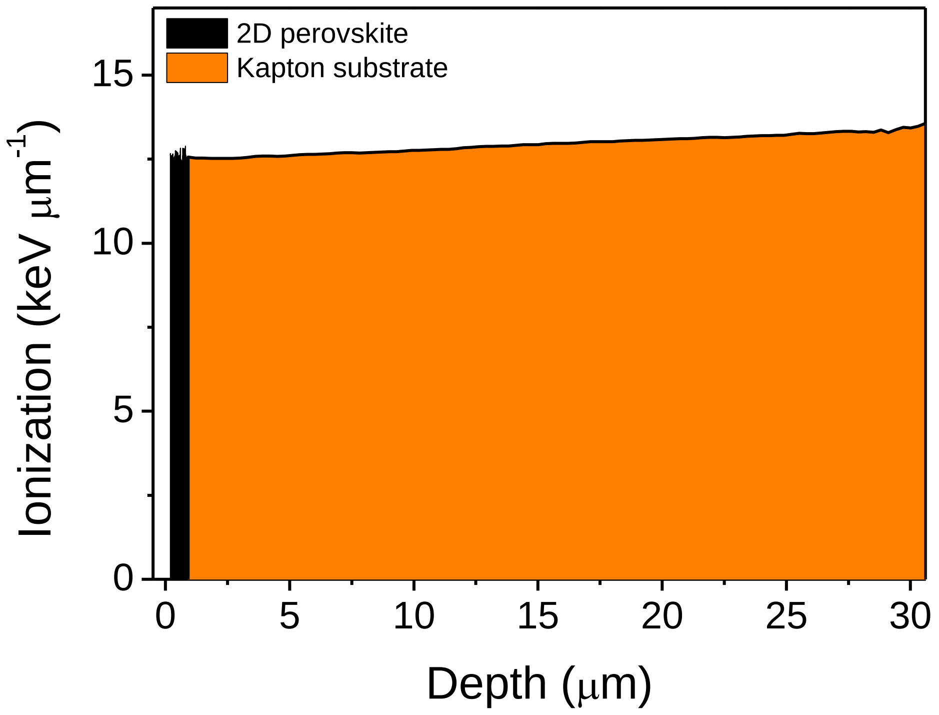

Figure S7. Monte Carlo SRIM simulation for the penetration of the protons in the detector. Each 5 MeV proton passes through the 2D perovskite layer releasing 12 keV μm^{-1}.

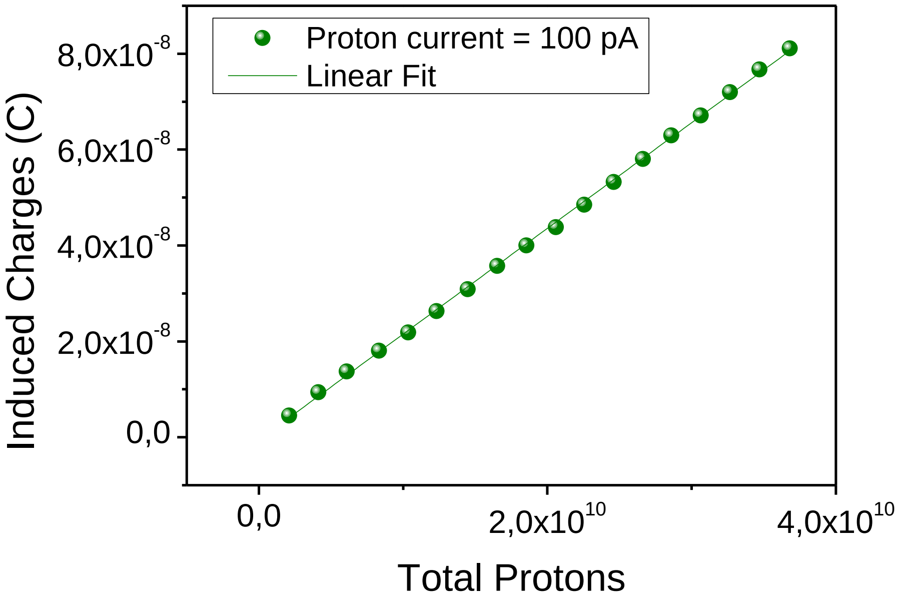

Figure S8. Dose Linearity curve obtained by integrating at different instant time the peak of current induced by proton beam at a fluence of $(1.2 \pm 0.1)\cdot 10^{10}$ H^+ s^{-1} cm^{-2}.

MATERIAL	ELECTRIC FIELD (V μm^{-1})	PROTON ENERGY (MeV)	SENSITIVITY	REF.
**$MAPbBr_3$ + $(PEA)_2PbBr_4$**	0.2	5	$(1.12 \pm 0.01)\cdot10^{-18}$ C $H^{+\ -1}$	[1]
**TIPGe-pentacene**	0.03	5	$(6.4 \pm 0.2)\cdot10^{-20}$ C $H^{+\ -1}$	[2]
**$MAPbBr_3$**	0.01	3	$(2.19 \pm 0.03)\cdot10^{-18}$ C $H^{+\ -1}$	[3]
**$CsPbCl_3$**	2	100-228	$4\cdot10^{-20}$ C $H^{+\ -1}$	[4]
**$(PEA)_2PbBr_4$**	0.2	5	$(4.25 \pm 0.02)\cdot10^{-18}$ C $H^{+\ -1}$	This work

Table S1. Comparison of proton detection sensitivities reported in literatures for devices based on other active layer materials.

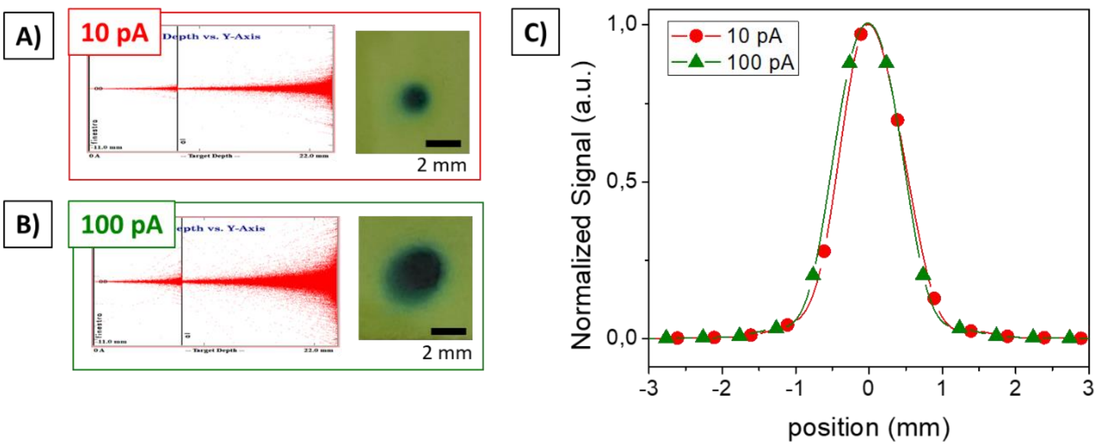

Figure S9. A and B report, on the left, the simulated transversal traces obtained by SRIM calculations and, on the right, the optical images of the beam spot on the radiochromic foils for the two conditions (i.e. 10 pA and 100 pA). (C) By normalizing the transversal trace of the events for the different number of protons coming from the source (i.e. 10^5 protons and 10^6 protons respectively for 10 pA and 100 pA), we obtain identical beam profiles confirming the fact that higher proton fluxes do not affect the proton beam shape.

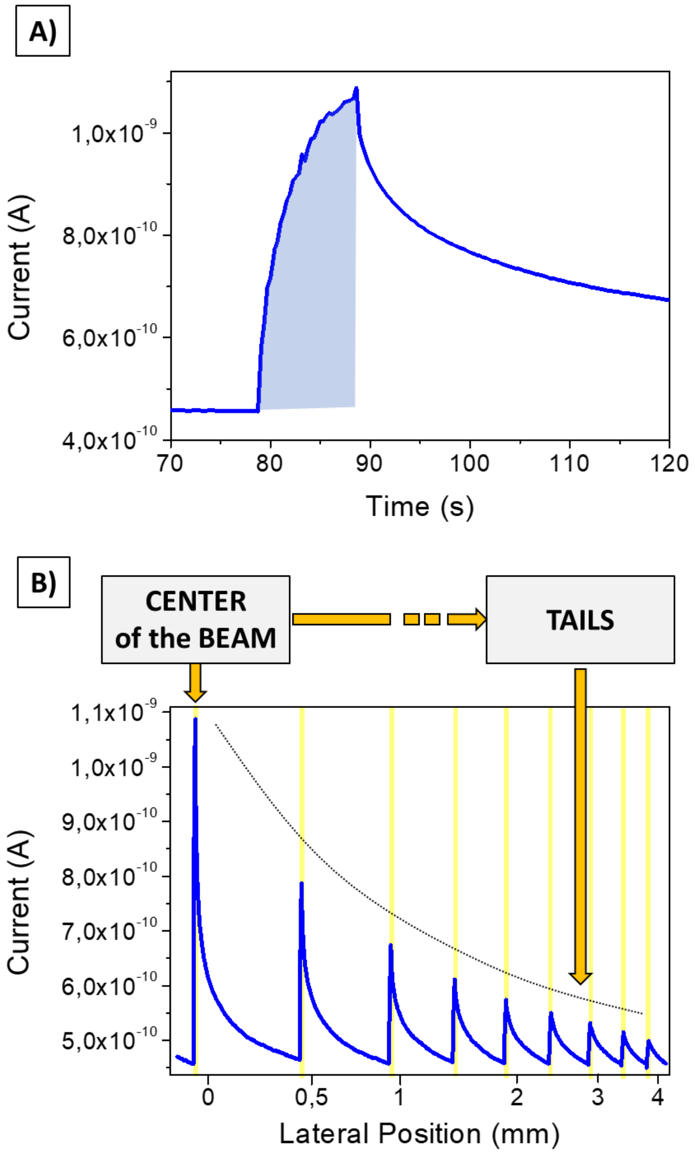

Figure S10. A) Dynamic response of the organic based detector polarized at 0.5 V (0.02 V µm^{-1}) and irradiated by 5 MeV proton beams at (9.0 ± 0.2)·10^{8} H^{+} s^{-1} cm^{-2} (10 pA) of intensity and 10 seconds of duration. The absorption of energy from proton beam provokes the increase of the current flowing in the device channel and the integral of the curve (blue shadow) indicates the proton induced charges collected at the electrodes. The slow response is the typical fingerprint of the photoconductive gain activation mediated by long-lived traps. (B) To reconstruct the beam profile, a single pixel has been moved transversally in front of the 5 MeV proton beam using a stepper motor (step = 0.5 mm). In each position, the signals of the detector induced by the beam at 10 pA have been acquired.

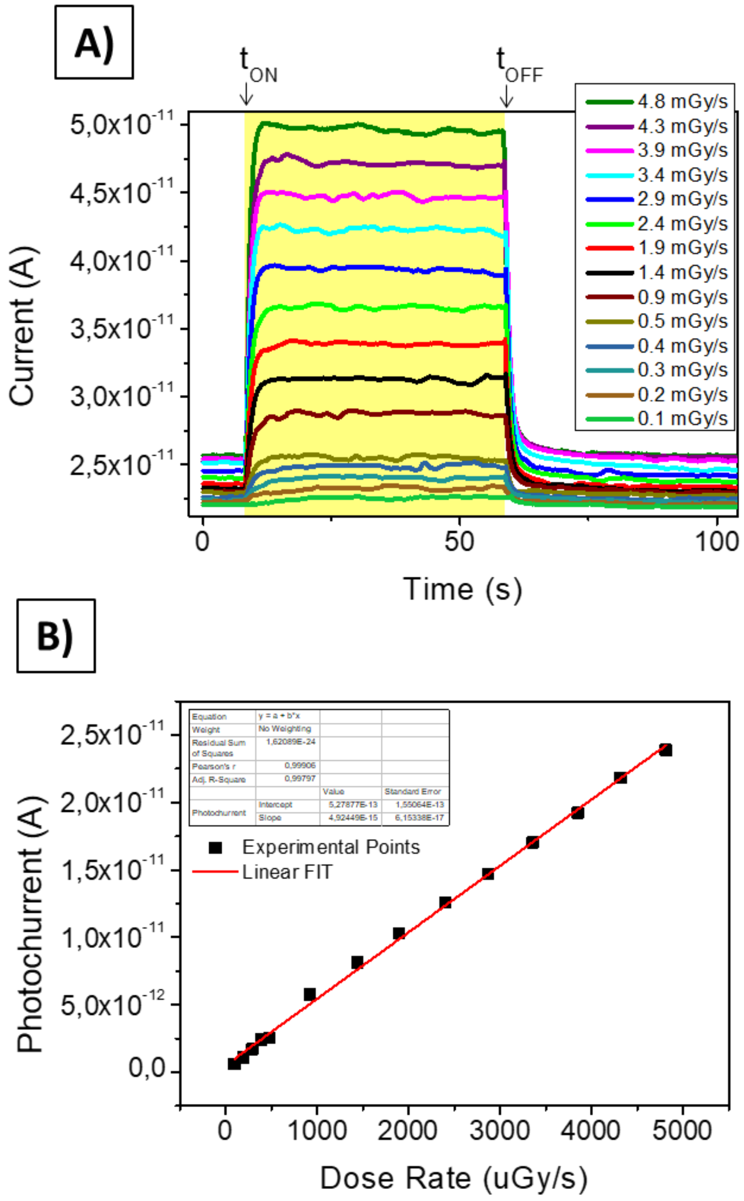

Figure S11. Characterization of the single pixel under X-rays. (A) Dynamic response of the 2D perovskite-based device polarized at 5 V (0.2 V μm^{-1}) and irradiated by X-rays produced by a W-target X-ray tube at 40 kVp anode voltage tuning the current in the 10-500 μA range. The duration of each irradiation cycle (yellow shadow) is 50 s and the dose rate values vary in the 0.1-5 mGy s^{-1} range. (B) The photocurrent induced by the absorption of radiation is calculated as the difference between the current flowing in the channel when the radiation is ON and the dark current present before the turning ON of the X-rays. The photocurrent scales linearly with the Dose Rate in accordance to what we previously reported [5]. The sensitivity can be expressed here as the slope of the linear fit and it results S_A = 123 ± 2 nC Gy^{-1} cm^{-2}.

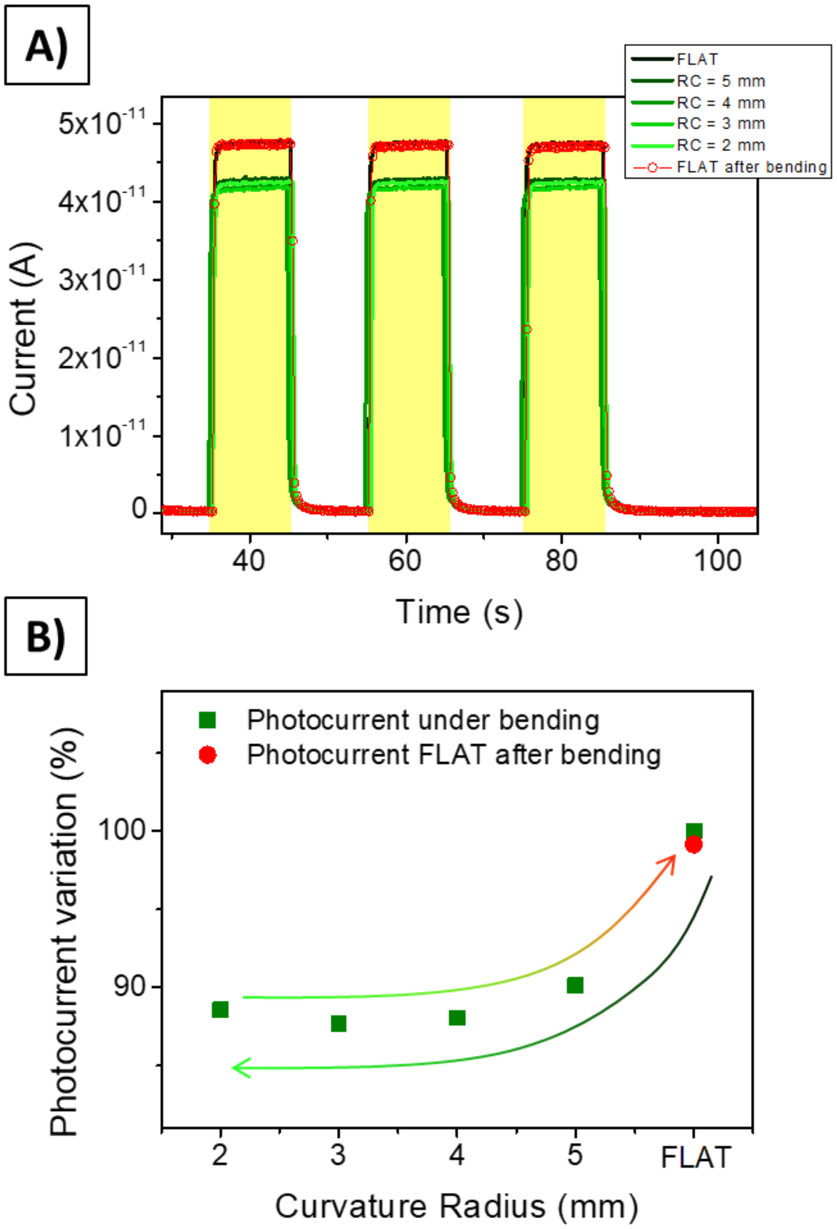

Figure S12. (A) X-ray induced signal under three subsequent irradiation cycles (10s X-ray ON, 10 s X-ray OFF, 40 kVp, 2.1 mGy/s, bias 5 V) while the samples were kept bent at different curvature radii in the range RC = [5 – 2] mm. (B) Photocurrent variation as a function of the curvature radius. The arrows indicate the first decrease of the photocurrent (i.e. 90% of the initial value) due to a gradual bending down to RC = 2 mm and the following complete recovery of the initial value once the sample has been placed back in the flat condition.

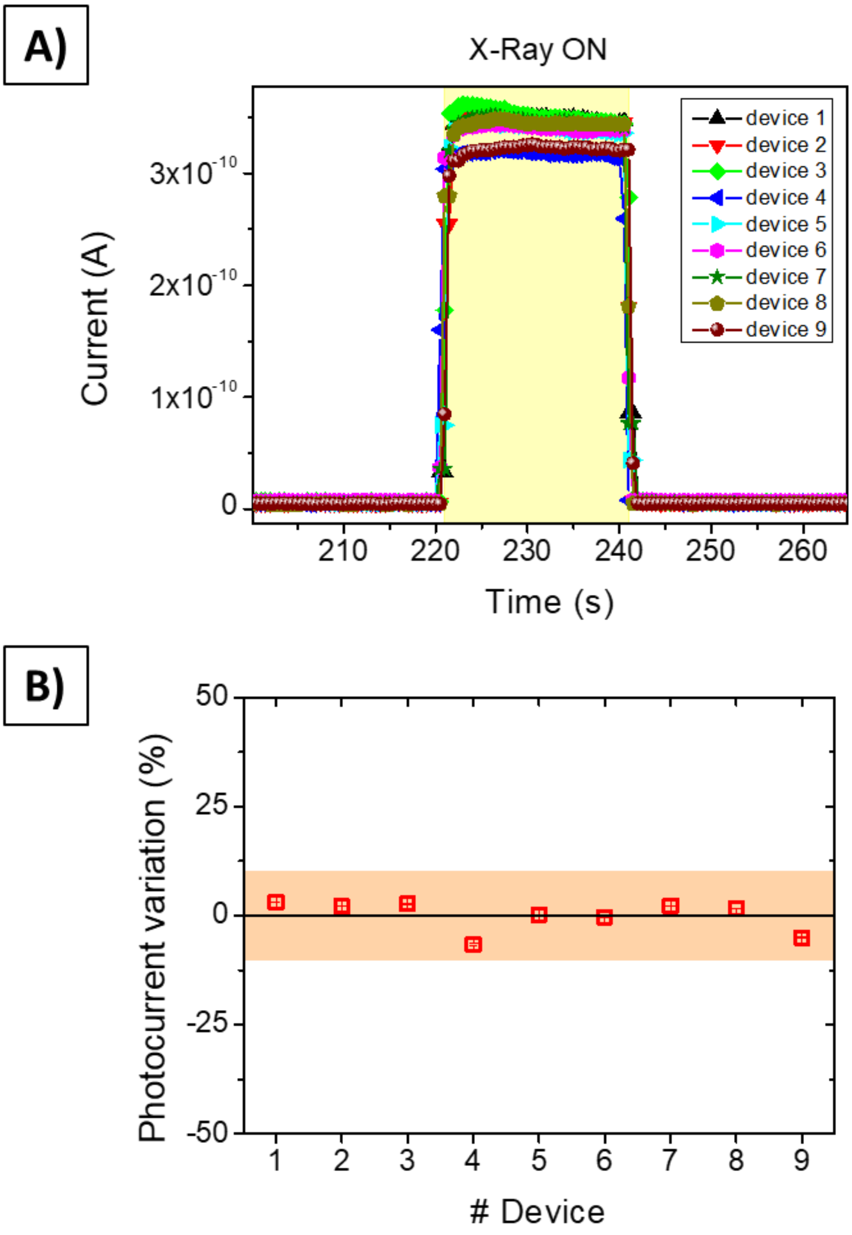

Figure S13. X-ray detection responses of the 9 pixels placed in the same array. (A) Dynamic response of the 9 pixels polarized at 5 V (0.2 V μm^{-1}) and irradiated by X-rays 20 second cycle (yellow shadow) produced by a W-target X-Ray tube at 40 kVp anode voltage. The dose rate impinging on each pixel is 8 mGy s^{-1}. (B) The photocurrent induced by the absorption of radiation is calculated as the difference between the current flowing in the channel when the radiation is ON and the dark current present before the turning ON of the X-Rays. The photocurrent variation for the 9 pixel is within 10%.

References

[1] L. Basiricò, I. Fratelli, M. Verdi, A. Ciavatti, L. Barba, O. Cesarini, G. Bais, M. Polentarutti, M. Chiari, B. Fraboni, *Advanced Science* **2022**, *2204815*, 1.

[2] I. Fratelli, A. Ciavatti, E. Zanazzi, L. Basiricò, M. Chiari, L. Fabbri, J. E. Anthony, A. Quaranta, B. Fraboni, *Sci Adv* **2021**, *7*, eabf4462.

[3] H. Huang, L. Guo, Y. Zhao, S. Peng, W. Ma, X. Wang, J. Xue, *ACS Appl Electron Mater* **2022**, DOI 10.1021/acsaelm.2c01406.

[4] M. Bruzzi, N. Calisi, N. Enea, E. Verroi, A. Vinattieri, *Front Phys* **2023**, *11*, 1.

[5] F. Lédée, A. Ciavatti, M. Verdi, L. Basiricò, B. Fraboni, F. Lédée, A. Ciavatti, M. Verdi, L. Basiricò, B. Fraboni, *Adv Opt Mater* **2022**, *10*, 2101145.
